# Screening Community-Living Older Adults for Protein Energy Malnutrition and Frailty: Update and Next Steps

**DOI:** 10.1007/s10900-019-00739-1

**Published:** 2019-09-30

**Authors:** Johanna T. Dwyer, Jaime J. Gahche, Mary Weiler, Mary Beth Arensberg

**Affiliations:** 1 Office of Dietary Supplements, National Institutes of Health, Bethesda, MD USA; 2 Jean Mayer USDA Human Nutrition Research Center on Aging, Boston, MA 02111 USA; 3 Department of Medicine and Community Health, School of Medicine and Friedman School of Nutrition Science and Policy, Tufts University, Boston, MA 02111 USA; 4 Abbott Nutrition Division of Abbott, Columbus, OH 43219 USA

**Keywords:** Protein-energy malnutrition, PEM, Undernutrition, Malnutrition, Screening, Frailty screening, Community-living, Older adults

## Abstract

Protein-energy malnutrition (PEM)/undernutrition and frailty are prevalent, overlapping conditions impacting on functional and health outcomes of older adults, but are frequently unidentified and untreated in community settings in the United States. Using the World Health Organization criteria for effective screening programs, we reviewed validity, reliability, and feasibility of data-driven screening tools for identifying PEM and frailty risk among community-dwelling older adults. The SCREEN II is recommended for PEM screening and the FRAIL scale is recommended as the most promising frailty screening tool, based on test characteristics, cost, and ease of use, but more research on both tools is needed, particularly on predictive validity of favorable outcomes after nutritional/physical activity interventions. The Malnutrition Screening Tool (MST) has been recommended by one expert group as a screening tool for *all* adults, regardless of age/care setting. However, it has not been tested in US community settings, likely yields large numbers of false positives (particularly in community settings), and its predictive validity of favorable outcomes after nutritional interventions is unknown. Community subgroups at highest priority for screening are those at increased risk due to prior illness, certain demographics and/or domiciliary characteristics, and those with BMI < 20 kg/m^2^ or < 22 if > 70 years or recent unintentional weight loss > 10% (who are likely already malnourished). Community-based health professionals can better support healthy aging by increasing their awareness/use of PEM and frailty screening tools, prioritizing high-risk populations for systematic screening, following screening with more definitive diagnoses and appropriate interventions, and re-evaluating and revising screening protocols and measures as more data become available.

## Introduction

As they age, most older adults want to retain their independence and remain in their own homes and communities for as long as possible. Life expectancy is increasing and arguably many older adults are healthier than their counterparts of comparable ages from past generations, but disability-free life expectancy still lags behind [1]. Because a growing number of older adults suffer from multiple acute/chronic diseases and disabilities, there is significant risk for both protein-energy malnutrition (PEM, often referred to as undernutrition or malnutrition) and physical frailty.

Screening for PEM/undernutrition and frailty is important because when these conditions go unrecognized and untreated, the risks of adverse outcomes and decreased functionality increase [2]. The rationale for screening for PEM and frailty risk together is due to the conditions’ common origins and signs/symptoms, their similar treatments and outcomes, and their frequent co-occurrence among both very old and lower-income minority groups [3, 4], as further described in Table 1. A systematic review assessing malnutrition and physical frailty found significant associations between the two conditions among 80% of the available studies of community-dwelling older adults [5]. The prognostic value of PEM and frailty screening tools used together was also good for predicting mortality among older patients with various medical and surgical conditions including acute heart failure [6], gastric cancer (post surgery) [7], and advanced colorectal cancer (receiving chemotherapy) [8]. Yet, although screening tools exist, they are inconsistently used as part of public health screening programs targeted toward community-dwelling older adults.

**Table 1:** Similarities between characteristics of protein-energy malnutrition (PEM)/undernutrition and frailty in older adults

Characteristic	Protein-energy malnutrition/undernutrition	Physical frailty
Prevalence in community-dwelling older adults	Not well documented but thought to be low for both conditions, may be higher in specific populations	
Subacute state exists	✓	✓
Measures available for screening in the community	✓	✓ Yes, but under development
Definition	Primary (modifiable) undernutrition due to inadequate intake of food to meet nutritional requirements	Disuse atrophy and age-related sarcopenia
Recognizable characteristics (phenotypes)	✓ BMI < 20 kg/m^2^ or 10% weight loss at follow up BMI < 22 in ages 70+ [33]	✓
Diagnosis	✓ Differential diagnosis needed to separate primary PEM/undernutrition from secondary PEM with other causes, and mixed primary and secondary conditions	✓ Differential diagnosis needed to separate primary disuse atrophy-related frailty from secondary causes of frailty and sarcopenia, and mixed primary and secondary conditions
Outcomes	✓	✓
Modifiable determinants	✓ Yes, for primary PEM; varies for secondary PEM due to etiology, and mixed primary and secondary PEM	✓ Yes, for poor nutrition, and disuse atrophy-related frailty; varies for others
Treatments	Helpful to treat both PEM and frailty together since they are strongly related to each other	

An additional challenge is that the screening tools currently used have dissimilar operating definitions for PEM and frailty, as well as different measures and cutoffs. Many are impractical for community settings and most are unvalidated, with high false positive rates and much misclassification. What is needed are data-driven approaches, combined with expert judgement, that focus on evaluating the validity and reliability of the available screening tools to identify PEM and frailty.

A half century has passed since the World Health Organization (WHO) summarized the criteria needed for effective screening programs [9]. Using this WHO framework, we reviewed published evidence on PEM and frailty screening of community-living older adults. This article describes the prevalence of these conditions and screening challenges, promising PEM and frailty screening tools that overcome some but not all difficulties in screening community-living populations, and next steps to promote systematic PEM and frailty screening and intervention to support healthier aging and decreased disability in the United States (US).

### Defining Effective Screening

Decades ago, the WHO established the following criteria (that are still in use today) for effective public health screening programs:

- There need to be agreed-upon definitions and objective criteria that can be used to describe the condition.
- The condition screened for must be a significant health problem and, if the condition is detected early by screening, effective treatments must exist.
- Validated tools to measure risk of occurrence must be available so those likely to be ill can be given priority for further assessment/treatment. Therefore, screening measures need to have satisfactory prevalence, criterion validity, sensitivity, specificity, and predictive validity.
- Populations most likely to benefit from screening must be identifiable and reachable.
- Screening tools must be suitable for the setting in which they are used (feasibility, performance, cost) and reflect changes in status that will result from effective interventions.
- Screening tools must be simple/noninvasive and a cost-effective standardized plan should specify the screening tool(s) and processes [9].

Developing community screening protocols starts with defining the elements of effective screening that include detection of a problem when early treatment can be more effective than after signs and symptoms develop, and identification of risk factors and use of this information to prevent/lessen the problem by modifying the risk factors [10].

Nutritional screening, the first step in the nutrition care process, involves the systematic identification of individuals at risk to establish whether a full nutritional assessment is needed [11]. Since the malnutrition screening tools described in this article focus on PEM, and not the entire panoply of all forms of malnutrition, they will henceforth be referred to as PEM screening tools. Screening for frailty–which has been viewed as a cornerstone of geriatric medicine–is also often undertaken to identify risk of adverse health outcomes [12].

Unfortunately, most available PEM and frailty screening tools have limitations for use with community-living older adults, since the tools were not developed/tested for effectiveness in this population or setting. Additional challenges to effective screening include:

#### Fragmented Healthcare Delivery

The capability and will to screen may be lacking because comprehensive assessment and intervention as an integrated part of overall healthcare delivery may not be available at the community level.

#### Limited Awareness that Problems Exist

Health professionals may be unaware that validated and feasible screening measures exist or believe PEM and frailty screening is outside their scope of practice [13, 14]. Fortunately, multidisciplinary education and advocacy efforts directed toward earlier intervention and treatment of preventable malnutrition and frailty are increasingly acknowledged [15].

#### Lack of National Survey Data

There is a lack of standard core measures on national surveys that could serve as benchmarks of PEM, other forms of malnutrition, and frailty prevalence or signals of risk. In the US Healthy People 2020 and other national health objectives, neither malnutrition nor frailty are singled out as conditions with targeted goals for older adults, in part because reliable prevalence estimates of these conditions are not available to assess the effectiveness of prevention and treatment interventions. Gahche et al. found no US national surveys of older adults that provided complete measures for both PEM and frailty risk screening, and thus they recommended adding measures for unintentional weight loss, loss of appetite, and grip-strength to national surveys to allow for risk screening for the conditions [16]. These simple measures provide the data needed to help monitor the nutritional health of older adults.

#### Failure to Prioritize Highly Vulnerable Groups in the Community for Screening

Efforts are most efficient and effective when concentrated on community groups whose demographics, residence, or health status suggest vulnerability and who may otherwise be overlooked. Most older Americans (93.5%) live independently in homes, apartments, or other community settings [17].

Table 2 summarizes how PEM and frailty screening compares to the WHO criteria for screening. Further considerations are described below, including common measures, prevalence, promising screening tools, and next steps for community-level PEM and frailty screening.

**Table 2:** Characteristics of PEM and frailty screening tools and programs for older persons in the community in comparison to World Health Organization criteria for screening for disease [9]

Criterion	Malnutrition	Frailty	Comments
Is the condition a significant health problem?	✓	✓	Both PEM and frailty are well-documented problems among older adults. The burden of disease is considerable on quality of life, morbidity, and mortality for both. Incident PEM in the community varies from 5–17% depending on the population [57]. Many malnourished older adults are frail, while a much smaller percentage of frail elders are malnourished [5].
Is the definition of the disorder and its characteristics agreed upon?	✓	✓	Sufficient agreement exists on the definition of PEM/undernutrition and physical frailty to make screening possible. However, there is still disagreement on other, broader definitions of both conditions.
Does a subacute state exist?	✓	✓	A subacute state exists for both primary PEM and frailty and for many of the conditions associated with these conditions, secondary to other health problems.
Do effective treatments exist?	✓	✓	Treatments exist for primary PEM/undernutrition and for age-related and disuse atrophy-related frailty although their effectiveness is still being studied.
Does earlier intervention lead to better outcomes?	✓	✓	The causes and natural history of both primary PEM and frailty vary. If the cause of PEM is simply lack of food or the cause of frailty is disuse atrophy or undernutrition, screening can help in early identification and interventions/treatments are likely to increase quality of life and decrease morbidity/mortality. When the causes are more complex and involve complications of chronic disease and/or other factors, recognizable stages may exist when screening can be applied, but interventions are more complex and may or may not singularly decrease morbidity/mortality. Other treatments, in addition to dietary measures alone, may also be needed, and outcomes may vary, depending upon etiology.
Is there a valid, suitable and acceptable screening tool available?	✓	✓	Tools are available but not yet agreed upon. While criterion validity is satisfactory, sensitivity, specificity and predictive validity need improvement.
Is there a defined population that can benefit for whom the screen should be recommended? Who are they? Can they be reached effectively?	✓	✓	There are populations of older adults living independently in the community who are at high risk and who can benefit from screening, such as those in congregate settings (including senior centers), home-delivered meal programs, assisted living facilities, adult day care centers, ambulatory health care facilities, and residential care facilities. There is some overlap between those who are at high risk of PEM (those receiving home care appear to be most at risk) and high risk of frailty, so it is important to screen older adults for both conditions.
How will screening be delivered?	✓	✓	Generally, must be delivered in nonclinical settings in the community by allied health or social service personnel, or self-administered. Minimal time, cost, and equipment are important criteria.
Is the optimal interval between screening tests known?	✓	✓	Varies. Both conditions can arise rapidly, in 1–2 weeks, especially when illness or other acute events occur. In other instances, the conditions arise over months or years. Therefore, optimal intervals vary depending on an individual’s health and other factors.
Is the screening program cost effective, given the cost of publicity, testing, assessment, treatment and follow-up, compared to the cost if screening was not done?	?	?	Few studies have addressed this question. Screening for both conditions together is likely more cost-effective than either alone since many of the same components are used in screening for both conditions. Better estimates of the prevalence of the detectable and treatable preclinical phases of PEM and frailty are needed. It will be helpful to compare different screening tools in the same population, rather than attempting to invent new tools.
Are screening test characteristics satisfactory? (Validity, reliability and other test characteristics)	✓	✓	Sensitivity of PEM screening tools is generally satisfactory, but specificity and predictive validity are poor. Little available data on frailty screening tool test characteristics; sensitivity is generally satisfactory, but similar to PEM screening tools, those for frailty have poor specificity and predictive validity.
Are there agreed upon criteria for what represents an abnormal result?	✓ All agree malnutrition is abnormal	✓ All agree physical frailty is abnormal	There is some disagreement about the severity of weight loss and/or BMI that represents PEM. There is disagreement about whether cognitive and social frailty measures should also be included in frailty screening, in addition to physical measures.
Can the quality of testing with tool be guaranteed?	✓	Requires training	Physical frailty assessment using the Fried Frailty Phenotype criteria requires some equipment.
Is it feasible in community settings?	✓	✓	
Does it require special equipment?		✓	For Fried Frailty Phenotype score, hand-grip dynamometer is required.
Is self-report possible?	✓	✓	Yes, but self-report measures of PEM such as weight loss, height and weight and the FRAIL scale are not as accurate as those measured by observers.
Is the time to administer it reasonable?	✓	✓	5–15 min.
Is the cost low?	✓	✓	
Is training required for administration?	✓	✓	Some observer training is required, but it is minimal (an hour or less).
Are results accessible?	✓	✓	Ideally the diagnosis and measures used after screening the condition can be entered directly into electronic medical records. The availability electronically varies.
Is screening acceptable to those who are being screened?	✓	✓	Older adults are highly averse to the identification of both PEM and malnutrition more generally, but they make little distinction between malnutrition generally and PEM. They are also averse to frailty identification because of the connotations related to loss of independence, but older adults appear to be amenable to measures designed to prevent the conditions. Screening tools for both PEM and frailty need validation against a “gold standard” that is a meaningful health outcome criterion. Ideally, such a gold standard should also be something that patients value highly and that is important [58].
Is the screening tool practical/feasible?	✓	✓	Yes. PEM tool: 48 screening tools for PEM/malnutrition used with older adults were evaluated; criteria to rate each tool were based on published evidence of appropriate screening. The final tool evaluation consisted of three equally weighted domains; *validation,* (whether the tool was validated and the quality of the validity study) *parameters* (what was measured with evidence for inclusion of suitable parameters viewed by experts as suitable for identifying malnutrition in older adults*)*, and *practicability* (qualitative research on barriers and facilitators to malnutrition screening). Each domain contained a maximum of 15 points with a total overall weighted maximum score of 45 points [24, 25, 59] Frailty: formal comparisons between tools have not been made.

*Criterion validity* is assessed by comparison of the tool’s identification of risk with that obtained using a “gold standard” criterion such as subjective global assessment by a trained clinician. Good scores for rating *predictive validity* are sensitivity and specificity over 80%, an area under the curve (AUC) of greater than 0.8, correlation coefficient of greater than 0.75, kappa of greater than 0.6 and odds ratio (OR) or hazard ratio (HR) of greater than 3. Tools are considered to have poor validity if they have a sensitivity or specificity < 50%, AUC < 0.6, correlation coefficient < 0.40, kappa < 0.4 and OR/HR of < 2 [24]

### PEM Screening in the Community

Definitional challenges can be confusing both as to the type and treatability of malnutrition. In this article, the term older adult malnutrition refers solely to PEM or undernutrition (International Classification of Disease (ICD-10) codes E43, E44, E46, E64) rather than to nutritional disorders characterized by a broader classification that also includes overweight, obesity, and other excessive alimentation (ICD-10 codes E65, E66, E67, E68) [18]. This distinction is critical since treatments for under and overnutrition are different. Even when the definition of malnutrition is restricted to PEM/undernutrition, there is frequent disagreement on specific cut-points for identification and criteria used to measure it, although for some screening measures, such as body mass index (BMI) and weight loss over time, there appears to be relatively high consensus on cut-points.

Another challenge is whether screening should identify only risk of PEM/undernutrition that can be treated solely by dietary means (primary PEM), or whether it should also include PEM due to other causes that requires different treatments (secondary PEM), if it responds to treatment at all [19]. In the US and other Western countries, the primary cause of undernutrition is disease accompanied by such factors as inflammatory activity, comorbidities, and dependency [20]. The problem of separating out individuals with primary PEM (who are likely to require only nutritional interventions) is especially salient for community programs. Community-based providers typically do not have access to the clinical services needed for assessment, diagnosis, and subsequent treatment of secondary malnutrition. The European Union Joint Programming Initiative Knowledge Hub project on Malnutrition in the Elderly (MaNuEL) defines malnutrition as primary malnutrition–a condition that is treatable by dietary means [19]. Malnutrition indicators evaluated through MaNuEL include low BMI, previous weight loss, moderate and severe decrease in food intake, and for older adults aged > 65, combined BMI < 20 kg/m^2^ and/or weight loss. MaNuEL researchers concluded that the criteria used strongly affected prevalence and, thus, suggested it may be preferable to look at each criterion separately, as each may indicate a different nutritional etiology [21].

PEM/undernutrition were somewhat similarly considered in the 2012 consensus approach for diagnosing and documenting malnutrition in hospitalized adults that was published jointly by the Academy of Nutrition and Dietetics (AND) and the American Society for Parenteral and Enteral Nutrition (ASPEN). The approach described six characteristics of severe malnutrition in adult patients: weight loss, insufficient energy intake, loss of muscle mass, loss of subcutaneous fat, fluid accumulation, and diminished functional status, and it required the presence of two of these six criteria for a malnutrition diagnosis [22].

In contrast, the Global Leadership Initiative in Malnutrition (GLIM) recommends that any validated screening tool of those it lists can be used to screen for PEM [23]. However, most tools on the GLIM list were validated in hospitalized populations, and thus they are unsuitable for community settings without further validation, particularly because the prevalence of secondary malnutrition is typically much lower in the community than in chronically ill/hospitalized populations, and even if identified, resources for further assessment and treatment are often lacking. Another challenge with the GLIM approach is that aggregated measures taken with various heterogenous tools cannot be used for valid prevalence estimates. Moreover, in using many different screening tools, there is the risk of identifying different individuals at risk, even in the same population, since the tools’ measurement criteria vary.

### Prevalence

More needs to be known about the prevalence of PEM, as that will determine how costly community screening may be. Prevalence needs to be high among screened populations so that the relative costs of screening programs in relation to the number of true positive cases detected that are amenable to treatment are justifiable for decreasing or eliminating adverse health consequences. The documented prevalence of PEM in community settings depends on what measures are used, as was illustrated in a recent MaNuEL study that employed harmonized definitions of malnutrition to assess prevalence in over 5000 community-dwelling, older European adults [21]. About 4.2% had BMI < 20 kg/m^2^, between 2.3 and 10.5% (depending on the country) had weight loss, and severe decreases in food intake were reported by up to 9.6%. The prevalence of BMI < 20 kg/m^2^ and weight loss occurring together never exceeded 2.6%. Thus, the criteria used strongly affected prevalence estimates. In the MaNuEL group’s most recent report, the prevalence of PEM risk) was found to be 8.5% in European community settings. It was higher in adults > 80 years, in women, and in those with one or multiple morbidities, and differed by geographic location and the screening tool employed [20].

### PEM Screening Tools

Table 3 summarizes several common PEM screening tools (sometimes referred to as malnutrition screening tools), major components of the tools, and the items/types of questions employed. The validity of PEM screening tools for older adults in community settings was reviewed as part of MaNuEL. A scoring system that included ratings for validity, the parameters used (what was measured), and evidence the measures were suitable for detecting malnutrition in older adults was applied [24]. Thirty-six unique studies were found validating 20 different malnutrition screening tools. The authors concluded that due to poor validation study design and results, there was insufficient evidence to make strong recommendations for any of the malnutrition screening tools. However, they did identify SCREEN II (Table 4), initially developed in Canada where it is still widely used, as having the greatest evidence of validity in the community [25].

**Table 3:** Malnutrition screening tools: components/domains and measurement items/questions employed

Screening tool	Components/domains	Measurement items/questions
Body Mass Index (BMI)	*Physical*	Calculation derived from height and weight
Council on Nutrition Appetite Questionnaire (CNAQ) [61]	*Nutrition or diet* Appetite *Psychosocial or psychological* Sick or nauseated when eating	4 questions on appetite, eating satiety, food taste, number of meals/day 1 question on sadness/happiness level
DETERMINE Checklist (Disease, Eating poorly, Tooth loss/mouth pain, Economic hardship, Reduced social contact, Multiple medications, Involuntary weight loss/gain, Needs assistance in selfcare, Elderly years above age 80) [62]	*Nutrition or diet* Number of meals per/day Dietary intake (fruits and vegetables, milk) *Alcohol* *Physical* Tooth or mouth problems *Food security* *Polypharmacy* Physical Unintentional weight loss Change in Mobility	Illness or condition impacting food type and intake < 2 meals/day Few fruits or vegetables or milk products 3 + drinks almost every day Chewing and swallowing difficulty Food security Eating alone (Reduced social contact) Do you sometimes have trouble affording the food you need? Polypharmacy Lost or gained 10 lb in the last 6 months Are you sometimes physically not able to shop, cook, or feed yourself?
Malnutrition Screening Tool (MST) [63]	*Physical* Unintentional weight loss *Nutrition or diet* Appetite	One question on recent weight loss One question on decreased appetite
Malnutrition Universal Screening Tool (MUST) [64]	*Physical* Unintentional weight loss *Nutrition or diet* Decrease in intake	Unintentional weight loss (past 3–6 months); weight and height is measured or documented from health records (self-report if not possible to measure or health record not available) Acute disease and no nutritional intake > 5 days
Mini Nutrition Assessment (MNA) [65]	*Physical* BMI Unintentional weight loss Mobility difficulties Arm and calf circumference *Psychosocial or psychological* Psychological stress or acute illness Neuropsychological problems *Nutrition or diet* Recent change in intake Number of meals/day Dietary intake of selected foods Mode of feeding *Independence* *Polypharmacy* *Pressure sores or skin ulcers?* *Nutritional status* *Health status*	Involuntary weight loss (last 3 months) 1 question on mobility (scoring is based on 3 questions–able to get out of bed, wheelchair without assistance, able to leave home) 1 question indicating stress or severely ill recently 1 neuropsychological question (dementia, sadness) 1 question on food intake decline due to loss of appetite, digestive problems or swallowing difficulties At least 1 dairy product/day 2 + legumes or eggs/week Meat, fish poultry every day 2 + serving of fruits or vegetables/day Amount of fluid/day Determine whether person needs assistance or can eat without help Lives independently (not in a nursing home)? ≥ 3 prescriptions/day Self-reported view of nutritional status Self-reported view of health status compared to others of a similar age
Mini Nutrition Assessment Short Form (MNA-SF) [66]	*Physical* BMI Unintentional weight loss Mobility difficulties *Psychosocial or physiological* Psychological stress or acute illness Neuropsychological problems *Nutrition or diet* Recent change in intake	1 question on mobility (scoring is based on 3 questions—able to get out of bed, wheelchair without assistance, able to leave home) 1 question indicating stress or severely ill recently 1 neuropsychological question (dementia, sadness) 1 question on food intake decline due to loss of appetite, digestive problems or swallowing difficulties
SCALES (Sadness, Cholesterol, Albumin, Loss of weight, Eating, Shopping) questionnaire [67]	*Physical* Weight loss Mobility difficulties *Laboratory measures* Albumin Cholesterol *Food security* *Psychosocial or physiological*	0.91 kg (2 lb) body weight loss in 1 month or 2.27 kg (5 lb) in 6 months Problems with shopping Problems with feeding oneself Albumin concentration < 4 g/L, cholesterol concentration < 4.14 mmol/L Having sufficient money to buy and prepare food Sadness
Seniors in the Community Risk Evaluation for Eating and Nutrition questionnaire (SCREEN I) or (SCREEN II) [68]	*Nutrition or diet* Appetite Chewing and swallowing Food intake questions *Physical*	Appetite Frequency of eating Chewing difficulties Swallowing difficulties Diet restrictions Eating alone Money for food purchases (only SCREEN I) Vegetable and fruit consumption Meat and alternative consumption Use of milk and milk products Fluid intake Cooking difficulties Shopping difficulties Weight change (increase or decrease) Weight perception (only SCREEN II) Unintentional weight change (only SCREEN II)
Simplified Nutritional Appetite Questionnaire (SNAQ) [69]	*Nutrition or diet* Appetite	Four questions on appetite, eating satiety, food taste, and number of meals/day

**Table 4:** Description of Seniors in the Community: Risk Evaluation for Eating and Nutrition (SCREEN II) questionnaire [68]

Explanation	SCREEN II has 14 items asking questions about weight change over 6 months, appetite, and swallowing difficulty (coughing choking, and pain swallowing food/fluids), meal skipping and satisfaction with the quality of food prepared by others. Shorter versions with only three items are now available (weight loss, appetite, and swallowing difficulty).
Parameters	Weight change, appetite, swallowing difficulty, meal skipping, satisfaction with quality of food prepared by others.
Scoring system	✓
Rationale	Focuses on modifiable physical and psychosocial factors that may affect food intake in older persons.
Agreed upon definition and characteristics for what the condition is that is being screened for	✓
Criterion for risk	✓, No scoring specifically stated.
Prevalence using tool	✓
Validity (criterion, construct, predictive), reliability and other test characteristics	Earlier versions of the SCREEN I and SCREEN II instruments were validated using a definition of malnutrition of < 20 BMI kg/m^2^ or unintentional weight loss of 5–10% but they yielded poor results (sensitivity 31%, specificity 98%) suggesting that they were not appropriate for older community-living adults [70, 71]. Therefore, additional testing was done and sensitivity scored at 84%, specificity 62%, positive predictive value 84%, and negative predictive value 62% (at a cut point score of 53) on SCREEN II. Test–retest reliability and inter-rater reliability were all improved (intraclass correlation coefficient 0.83) [68]. *Criterion validity* of SCREEN II has been evaluated in validation studies among both Canadians and New Zealanders living in the community, sensitivity ranged from 84 to 90% and specificity ranged from 62 to 86% depending on the study [72]. Wham et al. tested SCREEN II against clinical assessment by a trained dietitian; in older community dwelling New Zealander octogenarians who were assessed as at low to high risk by dietitians with access to medical history, anthropometrics and intakes. SCREEN II scores assessed 12 months prior to the distribution of the questionnaire were significantly correlated (re = 0.76, p < 0.01) with dietitian risk ratings; using a new cutoff of < 49 (for high nutrition risk) sensitivity was 90% and specificity 86% [73]. *Construct validity* of 3 items (weight loss, appetite, and swallowing difficulty) on SCREEN II (14 items) and on SCREEN II abbreviated version (AB) (8 items) were tested in a mailing to octogenarian Canadian men living in the community who had been assessed earlier in the Manitoba Follow-up Study (a longitudinal study) and compared to current self-reports of health status (F = 14.7, P = 0.001), diet healthiness (ρ = .17, P = 0.002), and importance of nutrition in successful aging (p = 0.10, P = 0.03). All were significantly correlated with the 3-item score [74]. *Predictive validity* of 3 items from SCREEN II and SCREEN II AB were used in the large, longitudinal, population-based Canadian Community Health Survey of Healthy Aging. Participants were followed through acute care hospitalizations and death records. Using Cox proportional hazards models, at 2-year follow up those classified at nutritional risk had higher risks of acute care hospitalization (HR 1.2 95%; CI 1.1–1.4) and death (HR 1.6 95%; CI 1.3–2.0) after adjusting for confounders [75]. SCREEN II was also linked with mortality in another study [76].

The MaNuEL research framework is specific to older adults. In contrast, AND conducted a systematic review on the validity, agreement, and reliability of tools to screen *all* adults for malnutrition regardless of age, medical history, or physical location (care setting) using AND’s evidence analysis process [26]; 69 studies met their inclusion criteria. The SCREEN II was not evaluated in AND’s review. The Malnutrition Screening Tool (MST) (Table 5) was found to exhibit moderate validity, agreement, and reliability with Grade I (Strong) evidence. The evidence supporting other screening tools was reported as Grade II (Fair) [27]. AND’s draft position paper on malnutrition screening concluded “based upon current evidence, the Malnutrition Screening Tool (MST) should be used to screen adults for malnutrition regardless of their age, medical history, or location” [28].

**Table 5:** Description of the Malnutrition Screening Tool (MST) [63]

Explanation	MST has 2 self-reported items asking questions about unintentional weight loss and appetite; originally developed for use with adult hospitalized patients, now studied and used in a variety of settings.
Parameters	Unintentional weight loss (last 6 months), eating poorly because of decreased appetite.
Scoring system	✓
Rationale	Focuses on weight loss and appetite, modifiable factors that may impact on muscle loss and food intake in older persons.
Agreed upon definition and characteristics for what the condition is that is being screened for	✓
Criterion for risk	✓, Score of 2 or more indicates potential risk.
Prevalence using tool	✓
Validity (criterion, construct, predictive), reliability and other test characteristics	The Academy of Nutrition and Dietetics Evidence Analysis Library Nutrition Screening Systematic Review identified 20 diagnostic, validity, or reliability studies meeting their inclusion criteria for the MST to identify malnutrition risk in adults across care settings, acute and chronic medical conditions and ages; 16 were positive-quality studies and 4 were neutral-quality studies. The reference standards for assessing validity were several, including the MNA, SGA, patient generated SGA, and expert dietitian/nutritionist assessing status. Validity was judged by examining sensitivity, specificity, negative predictive value (NPV) (given the greatest weight to avoid missing cases), and positive predictive value (PPV). Seven studies were in the ambulatory care setting and none was in the community setting. For the ambulatory setting, there was only one study, conducted in Vietnam among 29 outpatients over 65 years of age with chronic obstructive pulmonary disease. The sensitivity of all ambulatory studies ranged from 38% [77] to 100% [63, 78] and specificity ranged from 69.5% [78] to 94% [77] based on 4 studies. PPV ranged from 40% [63] to 83% [77], NPV ranged from 65% [77] to 100% [63, 78], based on four studies. One ambulatory setting study reported interrater reliability between nurse vs researcher vs patient (K = 0.83, P < 0.001) [78]. *Criterion validity:* the MST has been widely validated in hospitalized older patients in both Europe and Australia and has also been validated in older adults in rehabilitation; no studies have assessed the validity in community-living older adults [25]. *Construct validity:* has been examined in hospitalized patients, no reported studies for community-living older adults [60]. *Predictive validity:* has been examined in hospitalized patients, no reported studies for community-living older adults [60].

### Frailty Screening in the Community

Frailty (ICD-10 CM code R54) is variously referred to as old age senescence, senile asthenia, and senile debility. Sarcopenia (ICD‐10‐CM M62.84) is closely related to frailty and malnutrition and is a condition involving age-related muscle wasting and/or an underlying disease if one is present [18].

Muscle mass is the biological substrate of physical frailty that leads to functional impairment. The operational definitions of frailty and the measures used to identify it are highly variable. One popular description is the Fried Frailty Phenotype which defines frailty based on physical frailty characteristics (weakness, decreased endurance, slow performance, exhaustion, and weight loss) that are unique and separate from disability and comorbidities alone. Using the Fried Frailty Phenotype, a score of three of the five measures present defines the individual as frail and a score of one or two as prefrail [29].

Other definitions view frailty as related to the accumulation of various deficits, such as mental, social, and physical deficits, rather than as a specific and distinct set of criteria [30]. In the accumulation of deficits models, severity of frailty is scored by the number of accumulated disabilities or comorbid conditions (up to 30 or more depending on the specific model).

### Prevalence

Although frailty seems to be common in later life, it is often poorly documented in clinical records. Because of the different definitions used, populations screened, and variable cut-off measures employed, prevalence rates vary widely between studies. Understandably, when frailty is measured based solely on physical measures (such as a low BMI and physical activity), the documented prevalence is lower than for frailty measured based on definitions that also include other dimensions [31]. In a systematic review of cross-sectional studies using various definitions of frailty, Colllard et al. evaluated the prevalence of pre-frailty and frailty in community-dwelling adults > 65 years in the US, and in other countries [32]. The reported prevalence in the community varied significantly, from 4 to 59%, with an overall weighted frailty prevalence of 10.7% (95% confidence interval (CI) = 10.5–10.9) in 21 studies totaling 61,500 participants. Frailty prevalence increased with age and was higher in women (9.6%) than in men (5.2%). Prevalence rates for sarcopenia–one component of physical frailty–are reportedly lower than for frailty. However, the true prevalence of sarcopenia is likely unknown. Similar to frailty, it is not yet routinely measured in community settings and when it is, different measures are used for detecting its presence making it difficult to consolidate data and establish accurate prevalence estimates.Consensus is gradually emerging as previous definitions are revised, at least in Europe [33].

### Frailty Screening Tools

The characteristics of an ideal or “best” frailty screening tool are similar to those already discussed above, including strong criterion validity, reliability, feasibility, low cost, and predictive ability. Multiple frailty screening tools exist (Table 6), although compared to PEM screening tools, fewer are valid/feasible and fewer consensus papers/large systematic reviews have been published. The predictive validity of frailty screening tools varies. Studies of very old nursing home residents in France found frailty measures to be related particularly to balance and ability to rise from a chair without assistance [34]. In a large British cohort study, low physical capability predicted future mortality risk both in those under and over 70 years of age, even when physical capability was not associated with comorbidities [35].

**Table 6:** Frailty screening tools: components/domains and measurement items/questions employed

Screener*	Components/domains	Measurement items/questions
The BRIGHT Tool (Brief Risk Identification for Geriatric Health tool) [79]	*Health status* *Physical* *Psychological or Psychosocial problems* Cognition	Self-reported health status 6 Physical questions (e.g., help getting around indoors, shortness of breath walking across room, difficulty (and need help) bathing/showering, dressing 1 Psychosocial question (depression) 2 Cognitive impairment questions (memory, decisions)
Frailty Index [80]	*Physical* Weight loss or gain Tooth or mouth problems Exhaustion *Nutrition and Diet* Appetite *Health status* Hospitalization (past 3 months) Emergency room (past 3 months) Other *Psychological or Psychosocial Problems* Physical health or emotional problems limiting social activities	Physical questions (difficulty getting in and out of bed, bathing) ± 10 lb in past 6 months 1 question on problems chewing or swallowing Self-reported health status, comorbidities, disabilities
Fried Phenotype or Physical Frailty Phenotype [29]	*Physical* Unintentional weight loss Weakness Poor endurance, exhaustion, Slowness Low physical activity	Loss of ≥ 10 lb in 1 year or > 5% of body weight Hand-grip strength using dynamometer Self-reported exhaustion Timed walk Self-reported physical activity
The Gerontopole Frailty Screening Tool [81]	*Physical* Unintentional weight loss Exhaustion or fatigue Slowness Cognitive *Level of independence* Healthcare providers’ initial assessment	Mobility difficulties in the past 3 months Weight loss in past 3 months (no weight specified) More fatigued in the past 3 months Slow gait speed Memory problems in the past 3 months Question on whether healthcare provider thinks their patient is frail
Groningen Frailty Indicator [82]	*Physical* Unintentional weight loss Disability Physical fitness *Polypharmacy* (or a measure of co-morbidity) *Psychological or Psychosocial Problems* Cognition Psychosocial	4 physical questions (e.g., walking outside, getting dressed) Loss of 6 kg in 6 months or 3 kg in 3 months 2 disability questions (impaired vision and hearing) Self-reported or rated physical fitness ≥ 4 medications 1 cognitive question (memory problems, dementia) 5 psychosocial questions (e.g., depression, isolation, anxiety)
Prisma-7 [83]	Physical Social	4 physical questions (health problems that limit activities, needing regular assistance, health problems that require person to stay at home, requiring stick, walker or wheelchair to get around) 1 question on social support
SARC-F (Strength, Assistance in walking, Rise from chair, Climb stairs, Falls) Screen for Sarcopenia [39]	*Physical* Strength Walking Rising from chair Climbing stairs Falls	5 physical questions (lift/carry 10 lbs, difficulty/needs aids walking across room, difficulty transferring from chair/bed, difficulty climbing flight of 10 stairs, number of times fallen in last year)
Simple FRAIL (Fatigue, Resistance, Ambulation, Illnesses, and Loss of weight), Questionnaire (FRAIL Scale) [36]	*Physical* Fatigue Resistance Ambulation Weight loss *Illnesses (Comorbidities)*	4 physical questions (Are you fatigued? Cannot walk up one flight of stairs? Cannot walk one block?) Have you lost more than 5% of your weight in the last 6 months? Do you have more than 5 illnesses?
Strawbridge Questionnaire [84]	*Physical* Unintentional weight loss *Nutriton and Diet* Appetite (loss) *Psychological or Psychosocial Problems* Cognitive Sensory function or disability	3 physical and balance questions (experience of sudden loss of balance, arm weakness, leg weakness, dizzy when standing) 1 question (unexplained weight loss) 1 question (experienced loss of appetite) 4 cognitive questions (memory problems, focus) 6 questions (difficulty reading, vision problems, hearing)
Tilburg Frailty Indicator [85]	*Physical* Unintentional weight loss *Psychological* or *Psychosocial Problems* Cognition Social *Comorbidities* *Health status*	8 physical questions (walking, balance, hearing, vision, strength in hands, tiredness) Loss of 6 kg + last 6 months or 3 kg last 1 month 4 psychological questions (memory, depression, anxious, coping) 3 social questions (living alone, social isolation/support) ≥ 2 diseases or conditions Self-reported health status

*All are questionnaires with the exception of the Fried Phenotype, which has both a questionnaire and physical measures

Two promising tools for frailty screening that are easy to use–particularly in high-risk settings in the community–are described below. Since both tools are based on self-reports, one unanswered question is whether individuals can report signs/symptoms validly and reliably enough to be helpful. The FRAIL scale (Table 7) is a short, self-administered questionnaire [36]. Although the FRAIL scale has fair validity and is feasible for community settings, it has a relatively low specificity; it overestimates the number of individuals who are frail in a population. Both the FRAIL Scale and the SARC-F (described below) were found to be useful screens for a stepped care approach to detecting frailty among older community residents of Hong Kong [37].

**Table 7:** Description of the Fatigue, Resistance, Ambulation Illnesses, and Loss of Weight (FRAIL) Scale screening tool [36]

Explanation	Five item self-report questionnaire with 1 point for each component (fatigue, resistance, ambulation, illnesses, and loss of weight)
Parameters	Fatigue, resistance (ability to climb a set of stairs), ambulation (ability to walk a block), illnesses (≥ 6) and loss of weight (> 5% loss in 12 months).
Scoring system	✓
Rationale	Focuses on self-report by older adults of likely components of physical frailty.
Agreed upon definition and characteristics for what the condition is that is being screened for	Fatigue: How much time during past 4 weeks have you felt tired? (1 = all of the time/most of the time, 0 = other Resistance: Have you had any difficulty walking up 10 steps alone without resting and without aids? 1 = yes, 0 = no Ambulation: Have you had any difficulty walking several hundred yards alone and without aids 1 = yes, 0 = no Illness: Have you had more than 5 illnesses out of these 11 (hypertension, diabetes, cancer, chronic lung disease, heart attack, congestive heart failure, angina, asthma, arthritis, stroke, kidney disease)? 1 = yes, 0 = no Loss of weight: 1 = weight decline of 5% or more in past 12 months, 0 = no
Criterion for risk	✓, scored 0 = robust, 1-2 = prefrail, and 3-5 = frail.
Prevalence using tool	✓
Validity, (criterion, construct, predictive), reliability, and other test characteristics	*Construct validity:* in a study of middle aged women, 10412 women were assessed; the construct validity of frailty as a predictor of depression (measured on a depression scale) and disability (assessed by needing help with daily tasks) was examined. The relationship between changes in self rated health and changes in the FRAIL score between 2 subsequent surveys 3 years apart was good; 16 years later Cox models were used for survival analysis. Frailty increased with age. Each component of the FRAIL score correlated with the total FRAIL score (r = 0.13–0.82, all P < 0.001). Women who were frail in 1998 had an increased likelihood of being depressed (OR, 2.77; CI 2.12, 3.63), or disabled (OR 6.87; CI 4.84, 9.77) in 2013. The HR for death was 2.01; CI 1.40, 2.87. Having a deficit in each of the 5 items at baseline also increased the chances of being depressed or disabled 15 years later [86]. In middle aged African Americans, the scale correlated significantly with IADL, a short physical performance battery, grip strength, and one leg stand tests among 703 participants with no ADL difficulties at baseline, and those measures plus gait speed in 883 who had no baseline ADL dependencies [87]. Face validity was determined by assessment of associations with age, and construct validity was determined by assessing associations with measures of disability (ADL and IADL). Concurrent validity as assessed by relationships with changes in self-rated health in a longitudinal study of 12,432 older Australian women surveyed up to 6 times (1996-2011); FRAIL scale was moderately correlated with disability (r = 0.4 for ALD and r = 0.5 for IADL) and slightly higher using a 6-point scale [88]. *Predictive validity:* being frail at baseline significantly predicted difficulties in ADL, IADL, and mortality. In middle age African Americans among 423 older adults with no ADL difficulties at baseline and among 528 who had no baseline ADL dependencies (after adjusting for baseline values for each outcome) prefrailty at baseline significantly predicted future ADL difficulties, worse one leg stand scores, mortality in both groups, and also IADL difficulties as well in the groups without ADL dependence [87]. When the Brazilian version of the FRAIL scale was compared to the Fried Frailty Phenotype in a cross-sectional study of 124 older adults, it was associated with fatigue, aerobic capacity and physical resistance but not with weight loss [89]. Mean changes in FRAIL scores decreased for Australian women who reported improvements in self-rated health between successive surveys by at least a mean of 0.0 (CI 0.01, 0.15) and increased in those who reported their health had declined by at least a mean of 0.64 (CI 0.57, 0.70) [88]. A systematic review and meta-analysis of prospective cohort studies examining mortality risk and its associations with frailty as defined by the FRAIL scale showed that the FRAIL scale effectively identified frailty and prefrailty as well as exhibited in a graded manner the association with mortality risk. The 4 studies available which calculated the area under the receiver operating curve ranged from 0.54 to 0.70. The random effects meta-analysis on 3 studies with adjusted HRs of mortality risk according to the 3 frailty groups (robust, prefrail, and frail) on the FRAIL scale showed that frailty and prefrailty were significantly associated with higher mortality risk than robustness (pooled HR. 3.53 95% CI 1.66, 7.49 P.001; pooled HR 1.75 95% CI 1.14, 2.70 P.01, respectively) [90]. Aprahamian et al. found that in work with the Brazilian adoption of the FRAIL Scale that physical performance (ambulation and resistance) items on the scale were strongly associated with higher age and dementia, whereas health status (fatigue, weight loss, and illnesses) were more associated with female gender and depression, suggesting two subdimensions of the scale and different pathways to frailty [91]. Dong et al. used a Chinese version of the FRAIL scale in 1235 older Chinese adults. Reliability/convergent validity was exhibited by kappa’s of 0.2–0.4 (P.001) of each item with its corresponding alternative measures that included a depression scale, the TUG test, 4-minute walking speed, polypharmacy and MNA SF. Diagnostic accuracy was good. It classified more individuals as frail (17%) than did the Fried Frailty Phenotype (4%). Internal consistency was low (Kuder Richardson formula coefficient 20–0.485). Test retest reliability over 1- and 2-week intervals was good; intraclass correlation coefficient 0.708 [92]. Woo et al. used the FRAIL Scale to screen 816 elderly Hong Kong residents to determine if it had prognostic value in a stepped care approach to identifying frailty/sarcopenia in residents living in the community (as part of a clinical evaluation that included a comprehensive geriatric assessment of those classified as prefrail or frail). Volunteers administered the FRAIL scale and SARC-F, a brief screen for mild cognitive impairment, and measures of blood pressure, BMI and grip strength. The prevalence of prefrailty was 52%, and of frailty 12.5%. Prevalence rose with age and was more common in women. Among the prefrail or frail, 43% had sarcopenia. Those screened at frailty or prefrailty risk were then given a complete geriatric assessment. Compared to the prefrail, the frail were less physically active, had more chronic diseases, were taking more medications, had more falls, rated their own health as poorer, had a higher prevalence of sarcopenia, and more ADL and IADL disabilities [37].

Sarcopenia is closely related to frailty and has been considered a precursor to the physical manifestation of frailty [38]. Morley et al. developed the SARC-F screening tool (Table 8) for rapid identification of sarcopenia risk [39]. The SARC-F has good test–retest reliability [40], and high specificity, but low sensitivity [﻿36﻿, 41]. It did have some prognostic value after discharge to home among elderly Japanese patients who had been hospitalized with cardiovascular disease [42]. Ida, Keneko and Murata recently reviewed articles from 1960 to date that included data on the sensitivity and specificity of SARC-F’s diagnostic criteria for sarcopenia in older adults. Seven studies with a total of 12,800 subjects met their study eligibility criteria. Overall, these studies achieved similar pooled results of sensitivity and specificity using definitions of both the International Working Group on Sarcopenia and the Asian Working Group for Sarcopenia. Because few studies were calibrated to the Foundation of the National Institutes of Health reference standards (which are based on appendicular lean mass) a meta-analysis was not performed on the aggregated data. Although the screening sensitivity of SARC-F was poor, its specificity was high [43]; thus, it may be an effective tool for identifying those who should undergo further assessment to confirm a sarcopenia diagnosis.

**Table 8:** Description of the Strength, Assistance in walking, Rise from chair, Climb stairs, Falls (SARC-F) screening tool [39]

Explanation	5 item self-report questionnaire with variable points for questions related to each component (strength, assistance, rising, climbing, and falling).
Parameters	Strength (difficulty in lifting/carrying 10 lbs) 0 = none, 1 = some; 2 = a lot or unable; assistance needed in walking across room, 0 = none, 1 = some, 2 = able to with aids or unable; rise from chair—how much difficulty, none = 0, 1 = some. 2 = a lot or unable; climbing a flight of 10 stairs—how much difficulty, 0 = none, 1 = some, 2 = a lot or unable; number of falls over past year, 0 = none, 1 = 1-3 falls, 2 = 4 or more falls.
Scoring system	✓
Rationale	Rapidly diagnose sarcopenia since it leads to disability, falls, and increased mortality due to loss of muscle strength and aerobic function.
Agreed upon definition and characteristics for what the condition is that is being screened for	✓
Criterion for risk	✓, Scores range from 0 to 10, a score of 4 or greater is thought to predict sarcopenia and poor outcomes.
Prevalence using tool	? Varies with the population; few community studies.
Validity, (criterion, construct, predictive), reliability, and other test characteristics	The SARC-F has good test–retest reliability [40], high specificity but low sensitivity [36, 41], and some prognostic value. Tanaka et al. used the SARC-F questionnaire to identify physical limitations and poor prognosis in 257 elderly Japanese patients with cardiovascular disease who were being admitted to hospital. They administered SARC-F as well as obtaining handgrip strength, usual gait speed, a short physical performance battery score and 6-minute walking distance test. The outcome was prediction of the first all-cause emergency readmission or all-cause mortality; 27% patients were assessed as frail using the tool, and those with more morbidities or who were older were more likely to be frail. Physical function was significantly poorer in those with high SARC-F scores (> 4) vs those under 4. SARC-F had prognostic value; it predicted adverse events after discharge, and those with higher SAFC-F scores had higher event rates than those with lower scores [42].

### Assessment of those Screened to be at Risk

Screening with standardized and well-validated tools identifies those at risk for PEM and/or frailty, but an assessment must be conducted to complete a definitive diagnosis, establish etiology, and plan and implement an intervention.

### PEM Assessment

PEM assessment measures as well as those for malnutrition in general often focus on changes in body composition which may result from many causes. The simplest cause is primary undernutrition uncomplicated by other factors and due to insufficient dietary intake (particularly protein and energy). The resulting atrophy of muscle tissue can be further exacerbated by a sedentary lifestyle and/or the aging process itself. More complicated causes of malnutrition include secondary malnutrition due to chronic or acute disease or injury with or without inflammation, or mixes of primary and secondary malnutrition.

The GLIM included inflammation in its definition of malnutrition because the presence of inflammation may vitiate nutritional efforts unless the inflammation is also treated. The criteria established by GLIM to diagnose, grade, and assess malnutrition include non-volitional weight loss, low BMI, and reduced muscle mass (all easily observable phenotypical physical characteristics) as well as measurements linked more closely to etiology (reduced food intake, inflammation, and disease burden). GLIM outlined a definitive diagnosis to consist of at least one phenotypical and one etiological criterion and recommended that individual patients should then be assessed more thoroughly to determine causality because such diagnoses will not all respond to the same interventions [23].

### Frailty Assessment

Neither PEM, frailty, nor scarcopenia screening yields a definitive diagnosis. Further clinical assessment is necessary to confirm the presence, causes, treatments, and interventions. In US community settings, it is rarely possible to screen for physical frailty using functional tests because of lack of time and equipment; thus, functional tests should always be included in frailty assessment in clinical settings. The comprehensive geriatric assessment (CGA) is often considered the clinical “gold standard” for assessing frailty, but it is very time consuming. Other frailty tools have been suggested as useful in clinical settings, yet at present there is little consensus about the best method for diagnosing frailty. In addition, like malnutrition, frailty is complicated by chronic/acute disease or injury that can affect the ultimate diagnosis and intervention, and sarcopenia (which can result from pathologies involved in both malnutrition and frailty) further confounds diagnoses.

Clegg, Rogers, and Young completed a systematic review of the diagnostic test accuracy of simple tools for identifying frailty in prospective studies of community-dwelling older adults. Three studies were identified, involving 3261 participants with a median frailty prevalence of 1.5% based on either the cumulative deficits frailty index or CGA, which were used as the reference standards. Several different screening tools were examined, including gait speed, timed up and go (TUG) test, and the PRISMA 7 questionnaire. The tools were highly sensitive for identifying frailty, but had very limited specificity, suggesting that they lacked accuracy as single tests to identify frailty [44]. Nevertheless, the tools may be useful. In 2019, Ahlund et al. studied 408 frail elders, mean age 85 years, with a high comorbidity burden who needed inpatient emergency medical care. They assessed aerobic capacity and muscle strength during patients’ hospital stays and 3 months later. Both higher aerobic capacity (measured by a 6 minute walk test) and muscle strength (measured by hand-grip strength) were associated with lower mortality at 1 year. Moreover, a change for the better in these variables over the first few months post-hospitalization was also identified as important [45], pointing to the need for effective screening tools and interventions in the community to help treat frailty.

### Community-Based Interventions for PEM and Frailty

A number of different community-based interventions exist for both PEM and frailty and can be accessed by various providers and service organizations. Systematic reviews on several effective interventions are described below.

### PEM

Just as critical as the diagnostic assessment, the selected interventions for PEM are important for effective clinical outcomes. Many of the original papers on the topic refer to malnutrition although upon inspection it is clear that the term refers more narrowly to PEM/undernutrition. PEM interventions vary in their ability to prevent or improve relevant and meaningful outcomes such as nutritional status, morbidity, functional status, and mortality [46]. As part of MaNuEL, quality assessments were conducted using Cochrane and GRADE criteria on 18 primary intervention studies taken from 17 systematic reviews. Correa-Perez et al. reported the overall quality of the evidence was very low due to risk of bias, small sample size, and heterogeneous outcome measures and populations and this precluded relevant meta-analyses (except for body weight and BMI measures). Based on their meta-analysis of the few studies comparing the effect of oral nutrition supplements (ONS) versus usual nutritional care on nutritional status (measured by changes in body weight and BMI) the researchers identified small gains in body weight after interventions, but changes in BMI or percent change in body weight were not evident. They also identified two randomized controlled trials that showed improvements in functional status (measured by TUG and activities of daily living) in the ONS treated group. The researchers concluded there is a clear need for well-designed, randomized controlled trials that follow standard criteria for reporting interventions on relevant outcomes for treating the condition in older people [46].

An evidence profile review on PEM/malnutrition was recently undertaken as part of the WHO Integrated Care for Older People (ICOPE) project which formulated recommendations for the prevention and management of undernutrition among older people in community and primary care settings. The WHO ICOPE workgroup concluded there is adequate, moderate-quality evidence to suggest that ONS with or without dietary advice improves the nutritional status of undernourished older adults and that for older adults at risk for it there is adequate, but low-quality evidence to suggest that ONS with or without dietary advice may improve nutritional status [47].

These results are similar to those from a recent systematic review of randomized clinical trials of nutritional interventions (provision of dietary counseling and/or ONS) in older adults at risk of PEM. Interventions were viewed as effective if they improved nutritional status by an increase in energy intake of 250 kcal/day and a weight gain of at least 1.0 kg. The intervention effect was significant for weight gain (odds ratio [OR] 1.58, 95%; CI 1.16, 2.17), but not for energy intake (OR 1.59; CI 0.95, 2.66). After stratifying by the type of intervention, the intervention effect was significant only for an increase in energy intake when dietary counseling was given in combination with ONS (OR 2.28; CI 1.90, 2.73). Therefore, for older adults at risk of PEM, they identified nutritional interventions had a positive effect on energy intake and body weight, and dietary counseling combined with ONS was the most effective intervention [48].

### Frailty

For frailty, the evidence is still inconclusive on the effectiveness of specific interventions (except for physical exercise, particularly strength-bearing exercise) for older adults at high risk of frailty in the community. A recent review considered 21 randomized studies (totaling 5275 older adults > 65 years who were prefrail/frail) involving interventions to prevent frailty progression compared to alternative interventions, usual care, or no care. Physical exercise programs both reduced and postponed frailty, but only when the programs were conducted with groups. Favorable effects on frailty indicators were also achieved with interventions that combined physical exercise with ONS, with cognitive training, or combinations of these treatments [49].

Just as frailty prevalence studies are confounded by the different operational definitions and measures used to identify it, so too are frailty intervention studies. A recent scoping review of interventions and policies aimed to prevent/reduce the level of frailty identified 14 studies (12 randomized controlled trials and two cohort studies) but because six different definitions of frailty were used in the studies, it was difficult to assess the combined effects of interventions. The interventions that significantly reduced the number of frailty markers present or the prevalence of frailty included all types and combinations of physical activity interventions (with and without nutrition supplementation and/or memory training), and pre-habilitation (e.g. physical therapy plus exercise and home modifications) [50].

In a systematic review of the effects of health care interventions on quality of life in the frail elderly, van Rijckevorsel-Scheele et al. screened relevant articles and found 19 intervention studies that assessed intervention effects on quality of life. Not surprisingly, the studies were heterogeneous in their design and involved many different interventions and, thus, the results were inconclusive with respect to the effects of exercise interventions on the quality of life for frail elders [51]. By limiting its focus, a systematic review of 46 studies (totaling 15,690 participants) that only analyzed randomized controlled trials/cohort studies with primary care frailty interventions, identified strength training and protein supplement interventions (that also included physical activity) as the best interventions, both in terms of relative effectiveness and ease of implementation [52].

### Both PEM and Frailty

The efficacy of interventions or treatment of individuals who were assessed as suffering from both PEM and frailty has also been studied. One critical factor in evaluating interventions for both together is whether those receiving the interventions were frail because they were undernourished and thus likely to benefit from nutritional interventions [4] versus being frail because of complex chronic disease and requiring appropriate medical treatment. Certainly, patients who are at risk of PEM should be screened for frailty since PEM may also play a significant role in the prevention and management of sarcopenia [53, 54] and equally important, patients who are at risk of frailty should be screened for PEM as a potential underlying cause contributing to their frailty.

All frailty criteria are affected to some degree by poor eating habits, and frailty itself may have a negative effect on eating (due to decreased appetite which is related to lower basal metabolic rate secondary to loss of muscle) and ultimately diminish nutritional status [55]. In a recent systematic review of the impacts of interventions on both nutritional and frailty status of vulnerable older individuals, diet (especially energy intake and overall diet nutritional quality) was identified as a helpful intervention. Yet, the efficacy of nutritional interventions in treating frailty could not be verified because most of the studies were cross sectional, and longitudinal outcomes were unavailable [56].

To date, the evidence on the role of nutrition interventions in preventing or reversing frailty consists of small studies of short duration; more studies are needed that are adequately powered to assess the effects of nutritional interventions in preventing and/or treating frailty [55]. The data are strongest for the combined interventions of dietary protein and physical activity as key anabolic stimuli for muscle protein synthesis. Although dose and duration effects are not yet clear, recommendations for adequate diets that ensure ample intakes of protein (perhaps also vitamin D, antioxidant nutrients, and long chain fatty acids) as well as a physically active lifestyle (with strength bearing exercise) can be made for all older adults to preserve their quality of life [54].

### Next Steps and Conclusions

Like blood pressure and other vital signs, simple screening measures for PEM and frailty are important indicators of health risk. Screening at the community level to identify and treat preventable malnutrition and frailty risk among vulnerable older adults is feasible. Screening for these conditions should be a part of routine health care for older adults living in the community. The way forward begins with awareness and education to ensure development of core competency in screening for such health risks as part of everyday clinical practice among all those who care for older adults.

Since PEM and frailty are closely interrelated in older adults, meaningful prevalence estimates and benchmarks are needed to evaluate the effectiveness of interventions and to make comparisons between international, national and local findings. Incorporating common core screening measures for malnutrition and frailty–such as unintentional weight loss, poor appetite, and hand-grip strength–will strengthen existing measures in national surveys of older adults to generate such estimates [16].

Using these same measures in screening tools that are valid, feasible, easy and inexpensive to administer, in concert with appropriate diagnostic, assessment, intervention and follow-up services, will go far in helping prevent and treat malnutrition and frailty. The AND workgroup highlighted that when data gathered with non-validated tools enter large databases alongside data from validated tools, it compromises accuracy and raises questions about the overall quality of screening processes [28]. The same could be said for frailty screening, a consistent approach and use of validated tools is necessary. As more data become available, it will be important to reevaluate screening tools and processes for both malnutrition and frailty screening and to modify recommendations as appropriate to ultimately support healthy aging in the community.
